# Protective effects and possible mechanisms of mesenchymal stem cells and mesenchymal stem cell-derived extracellular vesicles against kidney fibrosis in animal models: a systematic review and meta-analysis

**DOI:** 10.3389/fphar.2024.1511525

**Published:** 2025-01-03

**Authors:** Yuanchen Lv, Zibo Hua, Xiaomei Lu

**Affiliations:** ^1^ 1st Clinical Department, China Medical University, Shenyang, Liaoning, China; ^2^ Department of Pathophysiology, College of Basic Medical Sciences, China Medical University, Shenyang, Liaoning, China

**Keywords:** mesenchymal stem cell, extracellular vesicle, kidney fibrosis, mice, systemic review

## Abstract

**Introduction:**

The risk of kidney fibrosis is significantly elevated in individuals with diabetes, chronic nephritis, trauma, and other underlying conditions. Concurrently, human umbilical cord blood-derived mesenchymal stem cells (hUCB-MSCs) and their extracellular vesicles (MSC-Exos) have gained prominence in regenerative medicine. In light of these observations, we are undertaking a meta-analysis to elucidate the influence of hUCB-MSCs and MSC-Exos on kidney fibrosis.

**Methods:**

To identify eligible trials, we conducted a comprehensive search of the CNKI, PubMed, Web of Science and Wanfang databases from inception to 24 October 2022. Furthermore, the methodological quality of the included studies was evaluated using the Systematic Review Center for Laboratory Animal Experimentation (SYRCLE) risk-of-bias tool. Besides, the weighted standard mean difference (SMD) with a 95% confidence interval (CI) was calculated using the Review Manager 5.4 software. The Stata (12.0) software was employed to assess the impact of factors on outcome heterogeneity and publication bias in the study. A total of 645 related research studies were retrieved, of which 14 that involved 219 experimental animals were included in the study.

**Results:**

In comparison to the control treatment, treatment with Human UCB MSC and MSC-Exos was observed to significantly enhance renal function in animal models of kidney fibrosis. This was evidenced by a reduction in serum creatinine (Scr) levels (p < 0.00001) and blood urea nitrogen (BUN) levels (p < 0.00001), as well as reduction of CD68^+^ macrophages (p < 0.00001), TdT-mediated dUTP Nick-End labeling (TUNEL)+ tubular cells(p < 0.00001), α-SMA levels (p = 0.0009) and TGF-β1 (p < 0.00001). P < 0.05 is deemed to indicate a statistically significant difference. Alpha-smooth muscle actin (α-SMA) is a specific protein that is normally expressed in myofibroblasts. The term “CD68+ macrophages” refers to macrophages that express the CD68 protein on their cell surface. Both macrophages and myofibroblasts have been linked to the development of kidney fibrosis. In this study, the quantity of CD68^+^ macrophages and α-SMA was employed as a means of gauging the extent of renal fibrosis. Additionally, transforming growth factor beta 1 (TGF-β1) is a significant cytokine implicated in the pathogenesis of kidney fibrosis. TUNEL-positive tubular cells represent tubular cells undergoing apoptosis. It is hypothesized that this may result in a reduction of tubular apoptosis and a delay in kidney fibrosis, due to the inhibition of the transformation of macrophages into myofibroblasts (MMT) and the disruption of the kidney fibrogenic niche.

**Conclusion:**

The principal findings of this preclinical systematic review indicate that hUCB MSC and MSC-Exos have a substantial protective impact against kidney fibrosis. Kidney transfer remains the final option for traditional renal fibrosis treatment. The lack of donors and high cost make it challenging for many patients to access appropriate treatment. Although this study still suffers from three shortcomings: sample size, methodological consistency and translational challenges, the hUCB MSC and MSC-Exos have been demonstrated to reduce tubular apoptosis and inhibit fibrotic progression. The hUCB MSC and MSC-Exos offer a promising alternative due to their lower price and accessibility. Nevertheless, further high-quality studies are required in the future to address the methodological limitations identified in this review.

**Systematic Review Registration:**

Identifier INPLASY2022100104.

## 1 Introduction

Chronic kidney disease (CKD) is defined by persistent urine abnormalities, structural abnormalities or impaired excretory renal function suggestive of a loss of functional nephrons. (Romagnani et al., 2017).It is becoming a global disease that affects 10% of the global population now. (Kidney Disease: Improving Global Outcomes KDIGO CKD Work Group, 2024). Previous study have shown that the global median prevalence of chronic kidney disease is 9.5% (IQR 5.9–11.7). (Jadoul et al., 2024).CKD can progress towards end-stage renal disease (ESRD), a condition that requires renal replacement therapy, such as kidney trans-plantation or dialysis. (Jha et al., 2013). Kidney fibrosis, characterized by excessive deposition of extracellular matrix (ECM) that leads to tissue scarring, is the final common outcome of a wide variety of CKD (Li et al., 2022). Fibrotic changes can occur in the glomerulus or in the tubules (Reidy et al., 2014; Duffield, 2014), Rather than being distributed uniformly across the kidney parenchyma, early renal fibrotic lesions initiate at certain focal sites in which the fibrogenic niche is formed in a spatially confined fashion (Li et al., 2022). The fibrogenic niche is a sites where fibrogenic cells aggregate and contribute to fibrosis development.

However, due to the lack of obvious clinical symptoms during the early stage, there is a lag in clinical screening. So much so that many cases have entered an irreparable stage when the development of kidney fibrosis diagnosed. Therefore, in clinical practice, the repair of renal fibrosis tissue and function mostly relies on tissue reconstruction and replacement, which is related to the application of regenerative medicine (Ciccocioppo et al., 2019).Previously, hUCB MSC with extremely low differentiation levels that can be preserved for a long time have become a new approach in the suppression of tissue fibrosis and the reconstruction of fibrotic tissues (Brown et al., 2019). The application of hUCB MSC in animal experiments has been considered effective in treatment of liver and pulmonary fibrosis (Huang et al., 2021; Rayia, 2022; Wang et al., 2013). However, several recent preclinical studies and clinical trials have reported that the therapeutic effect of Exosomes derived from hUCB MSCs is exerted through the paracrine production of growth factors, chemokines, and cytokines (Deng et al., 2015; Wang et al., 2015; Yao et al., 2015). Due to anti-inflammatory and tissue repair properties, hUCB MSC and MSC-Exos is becoming a promising therapeutic tool and an increasing number of clinical studies started to assess the therapeutic effect of hUCB MSC and MSC-Exos in different diseases (Lotfy et al., 2023). There have been a lot of studies on hUCB MSC and MSC-Exos to reduce the degree of kidney fibrosis, especially the fibrosis caused by complications of diabetes and the development of fibrosis caused by chronic kidney disease. Therefore, we decided to implement a systematic review to gather more dependable information on hUCB MSC and MSC-Exos for effect on kidney fibrosis.

## 2 Materials and methods

### 2.1 Study registration

The Systematic Reviews and Meta-analysis was guided by Preferred Reporting Items for Systematic Reviews and Meta-Analysis(PRISMA).Moreover the meta-analysis was registered with the INPLASY database(INPLASY2022100104).No ethical approval was required given that all analysis were implemented based on previously published research.

### 2.2 Search strategy

Two author (Hua ZB; Lv YC) independently searched for animal studies that related to Human UCB MSC-Exos and kidney fibrosis in the following electronic database: PubMed, China National Knowledge Internet (CNKI), Web of Science, WanFang. Any disagreements in the course of the discussion were resolved through discussion with a third author. All animal studies published from the date of inception of the databases to July 2022 were searched without language restrictions.

MeSH terms and Boolean operators were employed to refine the search strategy. The main search term used were: (Blood* OR Fetal OR Cord OR Umbilical Cord Blood* OR Cord Blood* OR Umbilical OR Umbilical Cord) AND (Stem Cell* OR Mesenchymal OR Mesenchymal Stem Cell* OR Multipotent OR Mesenchymal Stromal Cell* OR Mesenchymal Progenitor Cell* OR Wharton’s Jelly Cell* OR Adipose Tissue Derived Mesenchymal Stromal Cell* OR Adipose Derived Mesenchymal Stem Cell*) AND (Renal* OR kidney*) AND (Fibrosis* OR Cirrhosis*) AND (Randomized controlled Trial). At the same time, in order to make the research data more sufficient as far as possible, some gray studies that can obtain data are also included in the research scope. In order to record data, all initially selected records are entered into Review Manager 5.4.1 by two independent authors (Hua ZB; LvYC)All records with discrepancies in this investigation are resolved after discussion by the two independent authors(Hua ZB; Lv YC).

### 2.3 Study selection and prespecified outcome

**FIGURE 1:**
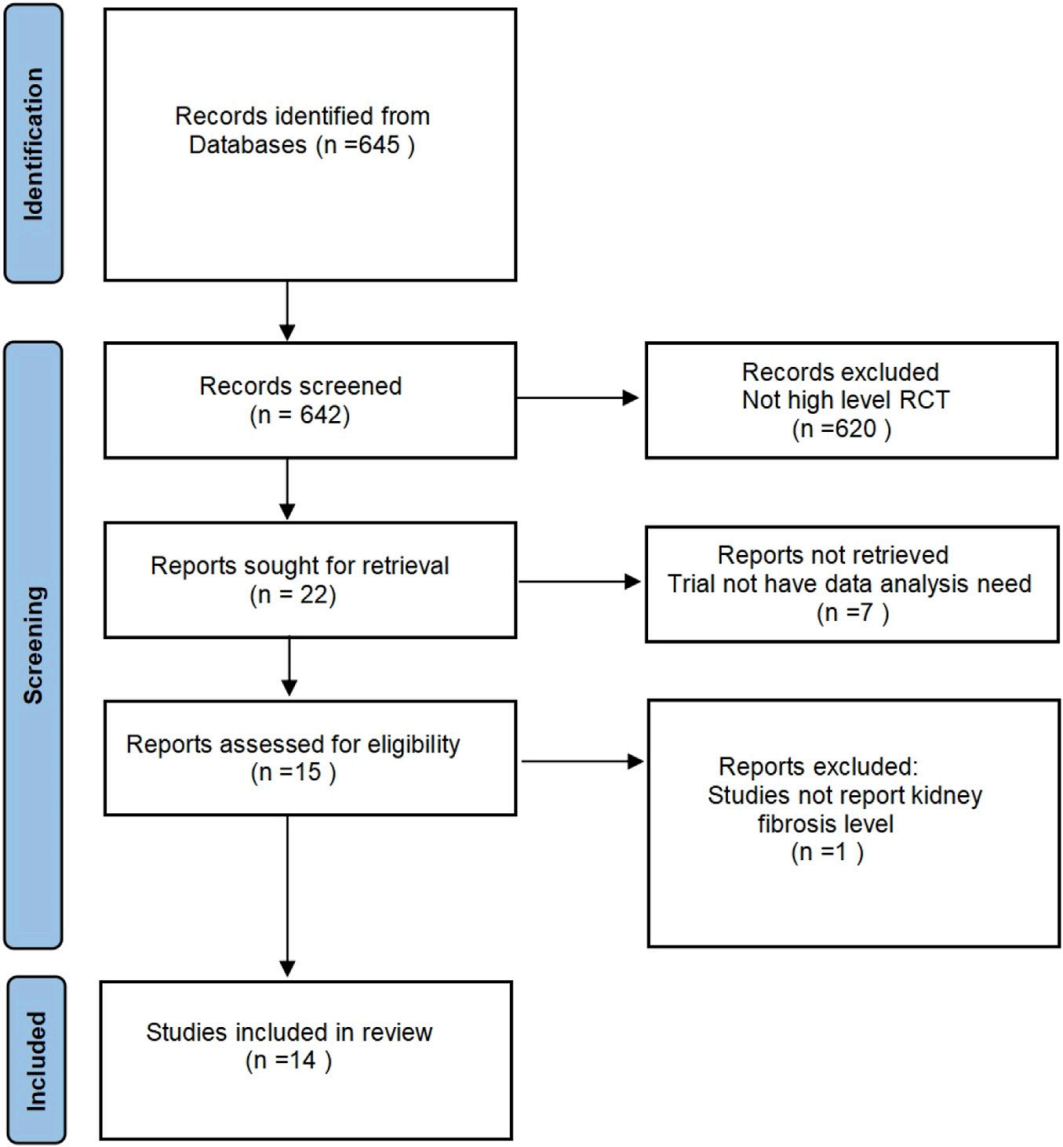
Flow diagram depicting the selection of studies.

The inclusion criteria of the study include: 1) experimental animals (rats and mice) in a healthy state; 2) animal experiments in the positive control group used by hUCB MSC, and there are no restrictions on the dosage and method of use; 3) the experiment is only limited to Randomized controlled trial; 4) the experimental results focus on the level of changes in the degree of kidney fibrosis. The specific search process is shown in Figure 1. This study mainly used the serum creatinine (Scr) level and serum Blood urea nitrogen (BUN) level of the control group and the experimental group to compare, but the experiment confirmed the existence of kidney fibrosis.

### 2.4 Data extraction and quality assessment

The two authors undertook the data extraction process, collating and analysing the data related to the definition of kidney fibrosis. We were able to obtain additional relevant data not provided in the original text by contacting the corresponding author and processing some image data.

In order to evaluate the potential for bias in the selected experiment, the SYRCLE quality assessment tool was employed to conduct bias testing. This process was overseen by two authors, who were responsible for monitoring the bias risk. The following aspects were considered in the evaluation: random sequence generation, allocation consideration, blinding of participants and personnel, blinding of outcome assessment, incomplete outcome data, selective reporting, and other sources of bias. In order to reduce publication bias, we conducted an exhaustive data collection and a comprehensive evaluation. No gray literature was identified in this research area. Each project is classified according to three risk levels: low, high, and unknown. In the event of any discrepancies or disagreements, these are resolved by independent authors.

### 2.5 Data analysis

To evaluate the effectiveness of each experiment, we used I^2^ to assess heterogeneity. I^2^ > 50% were considered to indicate significant heterogeneity. Regarding the use of models in different situations in data processing, this work uses Revman to first determine the p-value, and then determine whether to choose a fixed effects model or a random effects model. Fixed-effects models are typically employed for specific groups, whereas random-effects models are more commonly utilized for a broader range of groups. Consequently, in studies that encompassed both rats and mice, the random-effects model was selected, whereas the fixed-effects model was utilized for the remaining studies.

## 3 Results

### 3.1 Study selection and quality

A comprehensive search of four databases (PubMed, CNKI, Wanfang and Web of Science) yielded 645 articles relevant deemed to the topic under investigation. Following the reading of the titles and abstracts, the deletion of duplicates and the identification of randomized controlled animal experiments, 15 articles were retained. A detailed reading of the articles revealed that one article was excluded from further consideration as it did not present any results from *in vivo* research. Fourteen papers were ultimately retained, and data from 42 independent experiments were extracted for this article.

### 3.2 Characteristics of study

In this review, 11 studies used rats (9 SD, 1 Wistar, 1 thymus-free) and 3 used mice (2 C57, 1 Bal/BC). All 14 studies included male animals. 7 studies provided detailed information regarding the age of the experimental animals. The included studies employed 3 routes of administration (tail vein injection, direct intraperitoneal injection, left renal artery injection) and 4 modelling methods. (left renal ureter ligation, left renal hilum clamping, STZ intravenous induction, intraperitoneal injections) A total of 14 papers were included in the review, each of which utilized two distinct types of grafts: mesenchymal stem cells (MSCs) and MSC-derived exosomes (MSC-Exos). The results of the meta-regression analysis indicate that the type of graft (cell or exosome), the type of experimental animal, the mode of administration, the sex of the animal, and the method of modelling did not affect the heterogeneity of the results of the analyses, and thus a separate treatment was not required. It is, however, important to note that the method of modelling may have been a significant factor influencing the results of this study. The meta-regression results suggest that additional descriptive analyses should be performed to gain a deeper understanding of the impact of the modelling method on the results. The quantity of the administered drug was identified as a pivotal factor influencing the efficacy of the experiment, with a range of 10^4^–10^6^ for MSC and 10^4^–10^6^ for MSC-Exos in the included studies. No positive correlation was observed between the level of drug administration and either the species of experimental animal or the animal’s body weight. All studies included in this meta-analysis underwent ethical review by the relevant ethics committees.

### 3.3 Risk of bias and quality of the included studies

The risk of bias scores for the included literature obtained according to the SYRCLE tool are as Table1. Eight of the studies were rated as having a low risk of randomized sequences because they gave the exact manner and process of sequence allocation. All reports gave baseline characteristics between the two groups, and only one gave explicit allocation between different groups as sufficiently concealed. The experimental setup was consistent across the included studies, so we consider the placement of animals to be consistent with the principles of randomization. One study was identified as high risk because it did not report whether blinding was used in the investigation. Eight of the included studies referred to randomized outcome assessment, and four identified the use of blinding in outcome assessment. None of the studies had incomplete data, selective reporting of results, or bias from other sources.

**TABLE 1:** Risk of bias of included studies.

Study	A	B	C	D	E	F	G	H	I	J	Total
BoLiu2018	−	+	−	+	−	−	−	+	+	+	4
BoLiu2020	+	+	−	+	−	+	−	+	+	+	6
CamilaER2017	−	+	−	+	−	+	+	+	+	+	6
ChanJungLiang2015	−	+	−	+	−	+	+	+	+	+	6
ChengJi2020	−	+	−	+	−	?	−	+	+	+	4
EXiang2020	+	+	+	+	+	+	+	+	+	+	9
Guagyuanzhang2014	−	+	−	+	−	+	+	+	+	+	6
HongdeLi2020	+	+	−	+	−	+	?	+	+	+	6
J. H. Park, 2012	+	+	−	+	−	−	−	+	+	+	5
JingYu2017	+	+	−	+	?	−	?	+	+	+	5
TaoDu2021	+	+	−	+	−	+	−	+	+	+	6
YanHao2021	+	+	−	+	−	−	−	+	+	+	5
yihangYu2020	+	+	−	+	−	?	−	+	+	+	5
zhangxiangyu2020	−	+	−	+	−	+	+	+	+	+	6

(A) Sequence generation.(B) Baseline characteristics.(C) Allocation concealment.(D) Random housing.(E) Blinding ofexperimentalists.(F) Random outcome assessment.(G) Blinding of outcome assessors.(H) Incomplete outcome data.(I) Selective outcome reporting.(J) Other sources of bias.+:indicates low risk; −indicates high risk; ?indicates unclear risk.

### 3.4 Outcomes

#### 3.4.1 Indicators related to renal function levels

##### 3.4.1.1 Serum creatinine (Scr)

**FIGURE 2:**
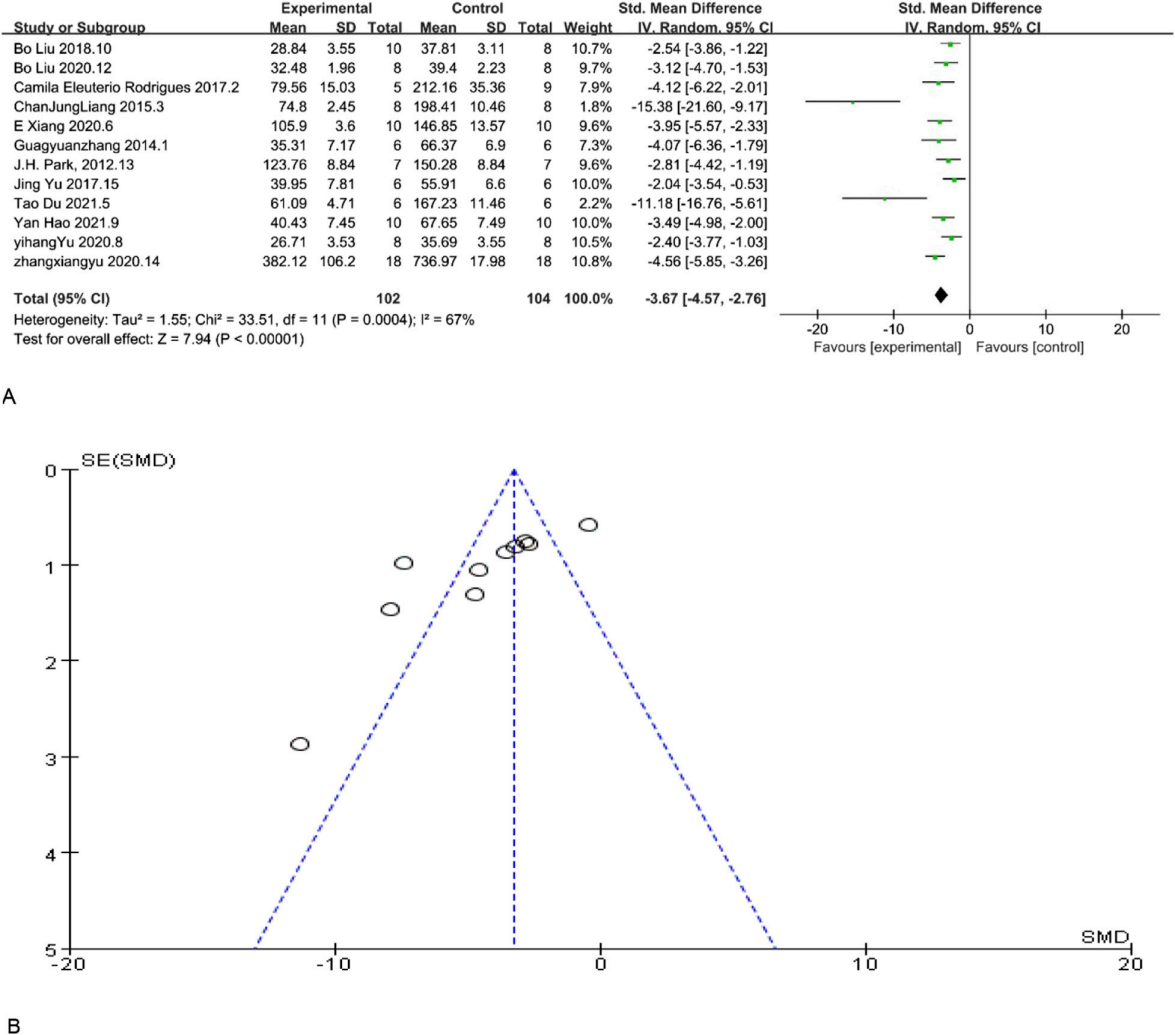
**(A)** Forest plot showing the pooled effect estimation of MSC and MSC-EXOS on Scr. **(B)** Funnel plot demonstrating the publication bias.

Scr is a significant indicator of renal function, and a reduction in its concentration indicate enhanced renal function. 12 studies included Scr as an indicator of renal function levels, and according to the overall analysis results showed that MSC and MSC-Exos significantly reduced Scr levels in the model animals, and renal function was overall higher in the MSC and MSC-Exos treated group than in the control group [n = 206,SMD = −3.67Cl (−4.57, −2.76), p < 0.05]. As illustrated in Figure 2, the heterogeneity analysis revealed a considerable degree of heterogeneity in the results (I^2^ = 67%). Figure 2 demonstrates the absence of publication bias.

##### 3.4.1.2 BUN

**FIGURE 3:**
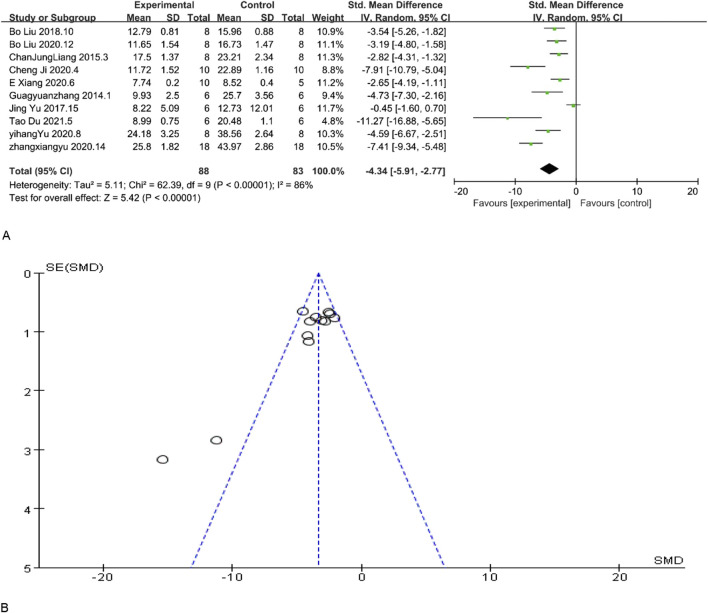
**(A)** Forest plot showing the pooled effect estimation of MSC and MSC-EXOS on BUN. **(B)**Funnel plot demonstrating the publication bias.

BUN is a crucial diagnostic tool for evaluating renal function. Lower BUN levels are typically indicative of enhanced excretory capacity of the kidneys and may also suggest optimal protein metabolism. The results of BUN demonstrated enhanced renal function. Among the included studies, 12 studies reported BUN levels, and according to the overall analysis results showed that MSC and MSC-Exos could reduce BUN levels in the experimental group, and the renal function levels of the MSC and MSC-Exos treated group were generally higher than those of the control group [n = 171, SMD = −4.34CI (−5.91, −2.77), p < 0.05]. As illustrated in Figure 3, the heterogeneity analysis revealed a considerable degree of heterogeneity in the results (I^2^ = 86%). Figure 3 demonstrates the absence of publication bias.

#### 3.4.2 Indicators of inflammation levels

##### 3.4.2.1 CD68

**FIGURE 4:**
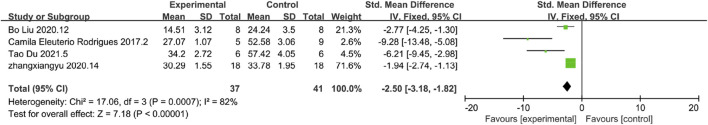
Forest plot showing the pooled effect estimation of MSC and MSC-EXOS on CD68.

CD68 is a key marker of macrophages, and an increase in the number of CD68-positive macrophages is indicative of heightened fibrosis. The results of CD68 demonstrated reduced inflammation response.A total of 4 studies reported the level of CD68, and according to the results of the analyses, it was found that the level of CD68 was significantly reduced in the MSC and MSC-Exos experimental group, which represented a lower level of inflammatory response in the experimental group [n = 78,SMD = −2.50CI (−3.18,−1.82), p < 0.05] As illustrated in Figure 4, the heterogeneity analysis revealed a considerable degree of heterogeneity in the results (I^2^ = 82%).

##### 3.4.2.2 TGF-β1

**FIGURE 5:**
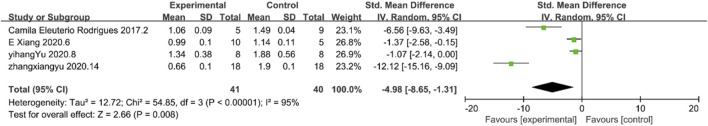
Forest plot showing the pooled effect estimation of MSC and MSC-EXOS on TGF-β1.

TGF-β1 is an important cytokine associated with kidney fibrosis. The results of TGF-β1 demonstrated reduced inflammation response. Four studies existed in the included literature that contained results submitted for TGF-β1 levels. According to the analysis, TGF-β1 levels were reduced to a certain level in the experimental group, which indicates the inhibitory effect of MSC and MSC-Exos on the inflammatory response in the experimental group, and the level of inflammation in the treated experimental group was lower than that in the control group. [n = 81, SMD = −4.98Cl (−8.65, −1.31), p < 0.05] As illustrated in Figure 5, the heterogeneity analysis revealed a considerable degree of heterogeneity in the results (I^2^ = 95%).

##### 3.4.2.3 α-SMA

**FIGURE 6:**
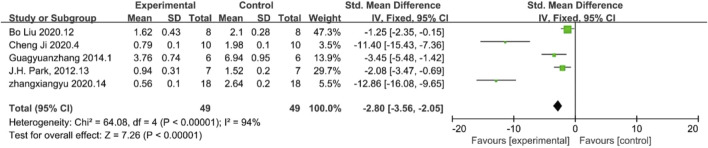
Forest plot showing the pooled effect estimation of MSC and MSC-EXOS on α-SMA.

α-SMA is a key marker for myofibroblasts which can cause kidney fibrosis. The results of α-SMA demonstrated reduced inflammation response. Five papers reported final levels of α-SMA levels, which, according to the analysis, were lower in the treated experimental group than in the control group, indirectly proving a certain level of suppression of inflammation levels in the experimental group compared to the control group. [n = 98,SMD = −2.80Cl (−3.−2.05), p < 0.05] As illustrated in Figure 6, the heterogeneity analysis revealed a considerable degree of heterogeneity in the results (I^2^ = 94%).

#### 3.4.3 Indicators of cellular change

##### 3.4.3.1 E-Cadherin

**FIGURE 7:**
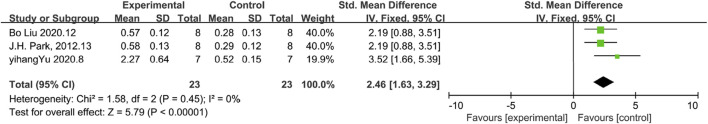
Forest plot showing the pooled effect estimation of MSC and MSC-EXOS on E-Cadherin.

E-Cadherin is an important expression of cellular function. The results of E-Cadherin demonstrated increased cellular function. Among the included studies, only 3 studies reported E-cadherin levels. According to the analysis, E-cadherin levels were higher in the experimental group than in the control group, which indicated a higher level of maintenance of cellular function in the experimental group than in the control group [n = 46, SDM = 2.46Cl (1.63,3.29),p < 0.05] As illustrated in Figure 7, the results of the heterogeneity analysis exhibit minimal heterogeneity (I^2^ = 0).

##### 3.4.3.2 Apoptosis detection by TUNEL method

**FIGURE 8:**
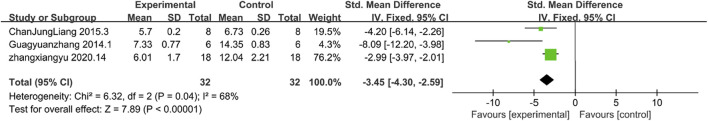
Forest plot showing the pooled effect estimation of MSC and MSC-EXOS on apotosis.

TdT-mediated dUTP Nick-End labelling (TUNEL)-positive cells Indicates the level of apoptosis in renal tubular cells.The results of TUNEL demonstrated reduced apoptosis. Only three papers reported TUNEL results. According to the analysis, the level of apoptotic cells detected by the TUNEL method determined that the level of apoptosis in the experimental group was lower than that in the control group [n = 64,SMD = −3.45,Cl (−4.30, −2.01),p < 0.05] As illustrated in Figure 8, the heterogeneity analysis revealed a considerable degree of heterogeneity in the results (I^2^ = 68%).

## 4 Discussion

### 4.1 Efficacy

Our search revealed that this is the first meta-analysis focusing on MSCs and MSC-Exos impact on kidney fibrosis and mechanisms of human umbilical cord blood mesenchymal stem cells on kidney fibrosis. The study included 14 studies comprising 219 experimental animals, distributed between experimental and control groups. In particular, we anticipated that there might be a considerable degree of heterogeneity across the studies prior to conducting the analysis, due to the presence of a number of variables inherent to the diverse experimental designs, including animal species, treatment duration, route of administration, modelling method, graft type, and so forth. Following the administration of treatments to the various groups, it was evident that a considerable degree of heterogeneity existed across the different analytical approaches. In order to ascertain the source of heterogeneity and to determine whether further analyses could be performed, a meta-regression analysis was conducted for the following variables: type of grafts (MSC or MSC-Exos), treatment duration, modelling method, drug administration method, and experimental animal species. The results of this analysis demonstrated that none of the aforementioned factors were a significant source of heterogeneity (P > 0.05) and that further analyses were not required. The full results of this analysis can be found in Table 2.

**TABLE 2:** meta−regression analysis of type of grafts (MSC or MSC−Exos),treatment duration, modelling method, drug administration method, and experimental animal species.

Sources of heterogeneity	P>|t|	(95% Conf.Interval)	
Modeling Method	0.813	−47.87	39.39
Route of Administration	0.742	−59.04	44.95
Animal Type	0.876	−394.38	631.98
Time of treatment	0.985	−3.91	3.97
MSc or MSC-ex	0.392	−163.27	350.69

Nevertheless, a single regression analysis indicated that modelling modality might be a potential source of high levels of heterogeneity. However, overall regression analysis demonstrated that this was not the case. The following section presents an analysis of potential explanations for the emergence of significant inter-subject variability.

It is possible that different modelling approaches may result in renal fibrosis states that are similar but not identical. The three methods have induced varying degrees of renal injury, resulting in disparate levels of fibrosis progression and modes of development. The modelling modalities of left renal ureter ligation and left renal hilum clamping demonstrated a greater propensity for more severe renal fibrosis than the induction of STZ by intravenous and intraperitoneal injections. Consequently, the identical treatment is administered to animal models that have been created through disparate methodologies. However, the discrepancies in disease progression will inevitably result in discrepancies in treatment efficacy, thereby elevating the level of independent heterogeneity.

### 4.2 Mechanism indicator

#### 4.2.1 MSC and MSC exosomes attenuate kidney fibrosis by reducing apoptosis

In the context of kidney fibrosis, tubular epithelial cells (TECs) are typically lost as a consequence of apoptosis (Kang et al., 2015). Previous research has demonstrated that transforming growth factor beta 1 (TGF-β1) can induce apoptosis in renal tubular epithelial cells (Miyajima et al., 2000; Xu et al., 2012). Our research has substantiated that mesenchymal stem cells (MSCs) and MSC-derived exosomes (Exos) can diminish the concentration of TGF-β1 in the kidney. Additionally, our analysis has validated that MSC and MSC-Exs can reduce the number of TdT-mediated dUTP Nick-End labeling (TUNEL)-positive cells, which indicate apoptotic tubular cells. Consequently, we hypothesize that MSC and MSC-Exos mitigate kidney fibrosis by reducing apoptosis.

#### 4.2.2 MSC and MSC exosomes attenuate kidney fibrosis by disrupting the kidney fibrogenic niche

The term “fibrogenic niche” has been put forth to describe the distinctive tissue microenvironment that stimulates fibroblast activation in the context of organ fibrosis (Fu et al., 2017). Prior research has demonstrated that the fibrogenic niche is comprised of macrophages and a diverse array of other structural elements. The activation of the kidney fibrogenic niche can influence the activation of fibroblasts in specific locations (Li et al., 2022). It can be concluded, therefore, that the fibrogenic niche plays an important role in kidney fibrosis.

The results of our research demonstrate that hucMSC-exos has the capacity to reduce the number of CD68^+^ cells. CD68 is a crucial marker for macrophages, which constitute the kidney fibrogenic niche. It can therefore be surmised that a reduction in the number of macrophages may serve to attenuate the formation of a kidney fibrogenic niche, which in turn may lead to a reduction in kidney fibrosis.

#### 4.2.3 MSC and MSC exosomes attenuate kidney fibrosis by inhibiting macrophages-to-myofibroblasts transformation (MMT)

The transformation of monocytes/macrophages into myofibroblasts represents a significant phenomenon associated with kidney fibrosis (Wang et al., 2017). The literature indicates that the macrophage-to-myofibroblast transition (MMT) is a recently discovered extrarenal genesis for myofibroblasts (Meng et al., 2016a).

TGF-β1 is an important cytokine associated with kidney fibrosis. The TGF-β1-Smad3 pathway has been confirmed as a major pathway for the transformation of macrophages into myofibroblasts (MMT) (Chen et al., 2014). The TGF-β1 complex (comprising TGF-β1 and TGF-β receptor 2) activates TGFR1, which in turn induces the phosphorylation of Smad3 and Smad2. These then form new complexes with Smad4. These new complexes then enter the nucleus and activate the Src-centric gene network, thereby promoting the MMT process in the fibrosing kidney (Meng et al., 2016b). Our research has demonstrated that hucMSC-ex reduce TGF-β1 levels and inhibit the progression of kidney fibrosis.

α-SMA is a key marker for myofibroblasts. Both macrophages and myofibroblasts have been linked to the development of kidney fibrosis. The administration of MSC and MSC-Exs resulted in the attenuation of kidney fibrosis, which was accompanied by the inhibition of the MMT process. In active kidney fibrosis, the number of CD68^+^ α-SMA + MMT cells correlates with the total α-SMA + myofibroblast population (Li et al., 2024). This study demonstrated that the reduction of α-SMA indicated an inhibition of the MMT process. Furthermore, the reduction of CD68^+^ cells was evidenced by a reduction in CD68^+^ α-SMA + MMT cells. MMT represents a substantial manifestation of kidney fibrosis, as evidenced by prior research indicating that MMT mediates the profibrotic effects of myofibroblast-derived exosomes on kidney fibrosis (Yu et al., 2024). Our research has demonstrated that hucMSC-ex has a clear inhibitory effect on α-SMA levels, which is indicative of its capacity to impede the macrophage-to-myofibroblast transition (MMT).

### 4.3 Limitation

Despite the greatest care and rigour being employed in accordance with the manual of systematic evaluation of animal intervention studies, the study is not without limitations.

Sample size: The sample size of 14 papers is insufficient for including *in vitro* study of the results. It is hoped that future studies will include a greater number of high-quality publications, thereby expanding the sample size and facilitating further research.

Methodological consistency: The utilization of randomization techniques, outcome measures and blinding is crucial for the quality control of the study; however, this was not adequately addressed in the included literature, which exhibited a high level of experimental design.

Translational challenges: The fibrosis mechanisms difference between species would reinforce the need for caution when extrapolating to human conditions. A further limitation of this study is that the data were derived from electronic scales rather than real data, which may have resulted in biased results.

Positive publication bias: Despite exhaustive efforts to identify and include gray literature and to obtain negative results, this study was unable to obtain valid data. Accordingly, despite the authors’ best efforts to conduct a comprehensive assessment, there may be a positive publication bias in this study. This may have resulted in an unduly optimistic assessment of the study’s findings and the exclusion of pertinent risks.

It is recommended that future research should focus on improving experimental methods, modelling methods and the quality of animal experiment reports in order to provide high-quality results.

## 5 Conclusion

Although the number of studies included in this meta-analysis is relatively limited and the quality of the studies is variable, our findings suggest that human MSC and MSC-Exos have an inhibitory effect on the progression of kidney fibrosis. The hUCB MSC and MSC-Exos are more cost-effective and accessible, this study illustrates the efficacy of the hUCB MSC and MSC-Exos in the treatment of renal fibrosis in animal models. This paves the way for further preclinical and potential clinical trials of this biologic, thereby increasing the potential for future use in patients with kidney disease. This study elucidates the efficacious inhibition of renal fibrosis by hUCB MSC and MSC-Exos treatment in animals, thereby establishing a foundation for prospective clinical studies and human trials.

## Data Availability

The raw data supporting the conclusions of this article will be made available by the authors, without undue reservation.
